# Multipronged heat-exchanger based on femtosecond laser-nano/microstructured Aluminum for thermoelectric heat scavengers

**DOI:** 10.1016/j.nanoen.2020.104987

**Published:** 2020-09

**Authors:** Sohail A. Jalil, Mohamed ElKabbash, Zihao li, Jihua Zhang, Subhash Singh, Zhibing Zhan, Chunlei Guo

**Affiliations:** a The Institute of Optics, University of Rochester, Rochester, NY, 14627, USA; b Changchun Institute of Optics, Fine Mechanics, and Physics, Chinese Academy of Sciences, Changchun, Jilin, 130033, China

**Keywords:** Heat-sink, Thermoelectric generators, Micro/nanostructuring, Emissivity

## Abstract

Femtosecond (fs) laser processing can significantly alter the optical, thermal, mechanical, and electrical properties of materials. Here, we show that fs-laser processing transforms aluminum (Al) to a highly efficient and multipronged heat exchanger. By optimizing the formed surface nano- and microstructures, we increase the Al emissivity and surface area by 700% and 300%, respectively. Accordingly, we show that fs-laser treated Al (fs-Al) increases the radiative and convective cooling power of fs-Al by 2100% and 300%, respectively, at 200 °C. As a direct application, we use fs-Al as a heat sink for a thermoelectric generator (TEG) and demonstrate a 280% increase in the TEG output power compared to a TEG with an untreated Al heat exchanger at 200 °C. The multipronged enhancement in fs-Al heat exchange properties lead to an increase in the TEG output power over a wide temperature (T) range (T>50°C). Conversely, a simple radiative cooling heat exchanger increases the TEG output power within a limited temperature range (T>150°C). We investigate the laser processing parameters necessary to maximize the spectral emissivity and surface area of fs-Al. Fs-Al promises to be a widely used and compact heat exchanger for passive cooling of computers and data centers as well as to increase the efficiency of TEGs incorporated in sensors and handheld electronics.

## Highlights

• We produced a novel heat-exchanger by creating nano/micro surface structures on Al via femtosecond laser processing.
• We increased the Al emissivity, surface area, radiative and convective cooling by 700%, 300%, 2100% and 300% respectively.
• We demonstrated a 280% increase in the TEG output power compared with an untreated Al heat exchanger at 200 °C.

## Graphical abstract

**Figure:**
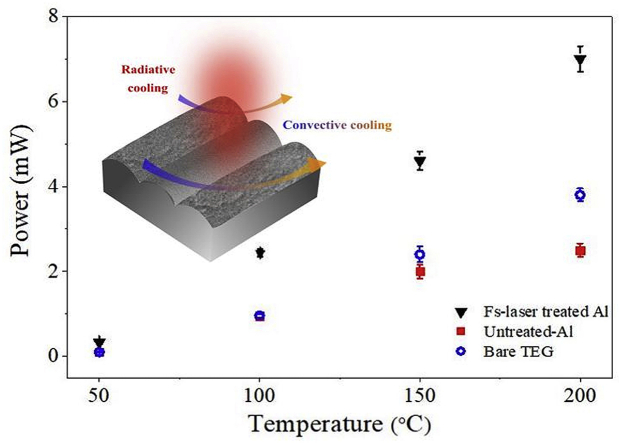

## 1 Introduction

A heatsink is a passive heat exchanger that can transfer the generated heat to a fluid, e.g., air, via convection, radiation, or conduction [1]. Realizing compact and multipronged heatsinks are essential for increasing the efficiency of electronic devices, e.g., computers and data centers. Research on passive cooling has been rekindled as it became necessary to minimize the carbon footprint of cooling, which accounts for 15% of global electricity usage [2]. In the context of clean energy applications, proper thermal management is necessary to maximize their efficiency. For example, photovoltaic and thermophotovoltaic cells require proper cooling to minimize the decrease in efficiency due to thermalization [[3], [4], [5]].

In particular, thermal management of thermoelectric generators (TEGs) is of significant importance. TEGs are heat scavenging devices that make use of the thermoelectric effect to convert a temperature difference into electricity [6,7]. While TEG modules are commercially available, they typically exhibit low efficiency but high reliability [6,8]. Thermoelectric power generation relies on the *Seebeck* effect, where the temperature difference across a thermoelectric module is directly converted to an electric voltage. Increasing the temperature difference $\Delta T=T_{h}-T_{c}$ generates larger electromotive force, where $T_{h}$ and $T_{c}$ are the temperatures of the hot and cold sides of a TEG module, respectively [6]. Consequently, TEGs can harvest energy during the day or night and operate indoors or outdoors by relying on any process that yields a temperature gradient. Increasing TEGs’ output power is crucial to expand their domain of applications beyond niche applications, e.g., industrial waste heat management and as power sources in extreme environments such as space probes [9,10]. It is expected that increasing the thermoelectric generated power of TEGs can open a wide range of applications particularly to operate autonomous, low energy consumption devices, e.g., wearable electronics and wireless sensors [11]. The temperature gradient across a TEG module is obtained by increasing $T_{h}$ [12], decreasing $T_{c}$ [13,14], or both [15,16]. Consequently, heat management of the TEG cold side is essential to maintain enough temperature difference, e.g., using a heatsink [2,17].

On the other hand, femtosecond (fs) laser processing can transform the optical, electrical, tribological, and mechanical properties of materials [18,19]. Through the formation of hierarchical nano- and micro-structures, fs-laser processing enabled the creation of black and colored metals [20,21], superhydrophobic and superhydrophilic surfaces [18,21,22], which found many applications, e.g., for solar absorber surfaces [[23], [24]], unsinkable metallic assemblies [25], and increasing the efficiency of incandescent lamps [26].

The cooling power (*Q*) of a heatsink attached to the cold side of a TEG is determined by the following equation [27]:

$$Q=Aq;\;q=h\left(T_{c}-T_{amb}\right)+\sigma \varepsilon \left(T_{c}^{4}-T_{amb}^{4}\right)\;\;\;\;\;\;\;\;\;\;(1)$$

where A is the heatsink area, q is the heat flux from the TEG cold side, which is determined by radiative, convective and conductive cooling, ε is the emissivity, σ is Stefan-Boltzmann constant, h is the overall heat transfer coefficient of the TEG cold side, and $T_{amb}$ is the ambient temperature. We consider the heatsink temperature to be ~ $T_{c}$ in our analysis which is a valid assumption when using a highly conductive heatsink, e.g., Al. The greater the cooling power of the heatsink (via radiative and convective cooling), the higher the temperature gradient and, in general, the efficiency of the TEG device.

**Fig. 1:**
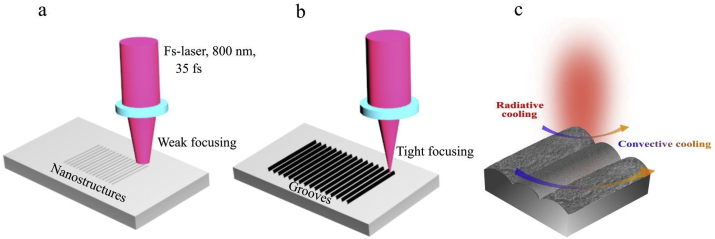
(a) Schematics of fs-Al surface via fs-laser processing at lower fluence. (b) Schematics of the fabrication processes of a multipronged heat exchanger by fs-laser processing of Al. (c) The formed surface structure increases Al emissivity, hence, radiative cooling power. In addition, fs-processing of Al surface increases its surface area, hence, its convective and radiative cooling power.

In this work, we present a novel heat exchanger based on the fs-laser processing of Al (Fig. 1b). The fs-laser processed Al (fs-Al) heat exchanger is multipronged with enhanced radiative and convective cooling capacity (Fig. 1c). The infrared (IR) spectral emissivity of fs-Al is controlled by controlling the size and density of the formed surface structures (Fig. 1a). We show that optimized fs-Al acts as an ultra-broadband perfect light absorber with near-unity emissivity for a wide range of temperatures. In addition, we optimize the laser processing parameters to increase the surface area of the fs-Al by controlling the depth and frequency of the formed grooves, thereby increasing the convective and radiative cooling power. We demonstrate experimentally a 280% increase in the TEG output power generated by a TEG with a fs-Al heat exchanger compared to a TEG with an untreated Al heat exchanger at 200 °C. More importantly, we show that enhanced TEG output power persists even at lower temperatures due to the increase in convective cooling. Consequently, fs-Al is an ideal heat exchanger with enhanced cooling power at a large temperature range.

## 2 Experimental section

### 2.1 Sample preparation

Polished Al foils were purchased from Goodfellow with the thickness of 0.2 mm and purity of 99.99%. Samples were cut into discs with size of 18 mm × 21 mm, degreased and washed with acetone and deionized water in an ultrasonic cleaner, respectively.

### 2.2 Fs-laser structuring

To fabricate controlled surface micro/nano structures on Al surface, we used an Astrella integrated Ti: Sapphire amplifier femtosecond laser from coherent as an irradiation source to deliver horizontally polarized pulse trains at the repetition rate of 1 kHz, with a central wavelength of λ = 800 nm and a pulse duration of τ = 30 fs. The maximum pulse energy delivered by the laser system is 7 mJ. The pulse energy was controlled using a combination of half-wave plate and a polarizer. The laser was focused by a lens of focal length 25 cm and incident at normal incidence. Bulk circular disks of Al were used as a target material. The samples were mounted at a computerized XYZ precision stage and processed by raster scanning the laser beam at different speeds across the sample. The target was placed ~300 μm before the laser focal plane to avoid strong damage. The density of the nano/microstructures is controlled by changing the laser fluence from 0.20 J/cm^2^-3.0 J/cm^2^. To increase the surface area at the microscale, interspacing between two adjacent scanned lines were changed from 100 μm to 160 μm. The laser-treated area for the TEG output power measurement in Fig. 4 is 18 × 21 mm^2^, and in Fig. 5 and Fig. 6 is 11 × 11 mm^2^.

### 2.3 TEG measurements

Commercial Bi–Te based TEGs (module of TE-MOD-1W2V–21S) with size of 18 mm × 21 mm were purchased from TEGprotm. Bi–Te based TEG power modules can continuously operate at temperatures as high as 230 °C. TEG measurements were performed on a hotplate at temperatures of 50 °C, 100 °C, 150 °C, and 200 °C. Al foils and fs-laser-treated heatsinks were mounted on the cold side of a TEG by a high-temperature conductive paste (Omega™). The hot side of the TEG was connected to the hotplate by the same paste. TEG output measurements were taken after the sample is connected, and the hotplate temperature became stable for at least 10 min. Temperatures is measured by an IR thermometer (FLIR TG 167). The output current and voltage of the TEG were recorded by a source meter (Keithley-2400), and the output power was calculated based on the measured current and voltage. Each measurement was repeated for at least four times.

### 2.4 Surface and optical characterization

The surface morphology of fs-laser-treated samples is analyzed by scanning electron microscopy (SEM) and three-dimensional (3D) laser scanning microscope. The SEM is a Zeiss-Auriga field emission operating at an accelerating voltage of 20 kV. The spectral scattering/reflectance of the samples were measured using Fourier transform infrared (FTIR) spectrometer, Bruker IFS 66/S FTIR spectrometer, equipped with an integrating sphere, where the measured range of the wavelength was 2.5–25 μm.

## 3 Results and discussion

**Fig. 2:**
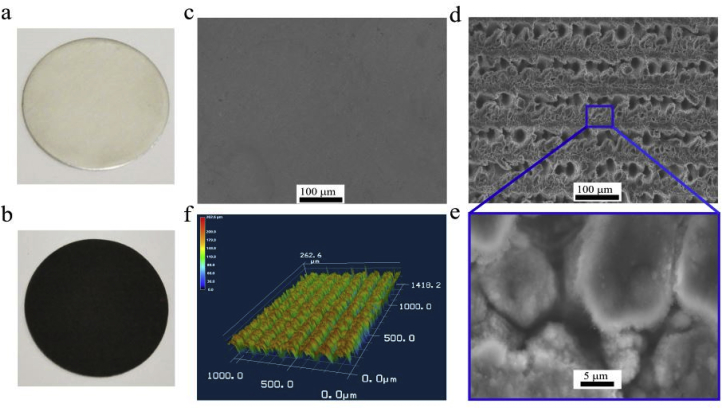
Photographs for (a) untreated Al and (b) femtosecond laser-treated Al (fs-Al). SEM images of (c) untreated Al, and (d) fs-Al (scale bar- 100 μm). (e) Magnified SEM image of fs-Al showing the formation of hierarchical nano/micro surface structures due to laser ablation (scale bar- 5 μm). (f) 3D profile of fs-laser-induced surface structures.

Fs-laser processing of surfaces can strongly modify the treated material properties. Fig. 2a and Fig. 2b shows photographic images of the untreated and fs-Al foils, respectively. By mere inspection (Fig. 2b), we can see that fs-Al turns into a super light absorber in the visible range [20]. Fig. 2c shows an SEM image of an untreated-Al. Following fs-laser processing (laser fluence *F* = 3 J/cm^2^ and scanning speed *V* = 0.5 mm/s), randomly distributed surface micro- and nanostructures form as shown in Fig. 2d and Fig. 2e. In addition, fs-laser processing drills the surface and creates ridges and grooves which increases the overall surface area at the microscale. Fig. 2f shows a 3D image of the same surface imaged in Fig. 2d using a laser scanning microscope. The optimum groove's depth is ~132 μm, and its number density is ~12000 grooves. m^−2^. Consequently, the surface area of the fs- Al is ~300% larger than that of untreated Al. The surface area increase is due to the area of the two side walls for each groove.

**Fig. 3:**
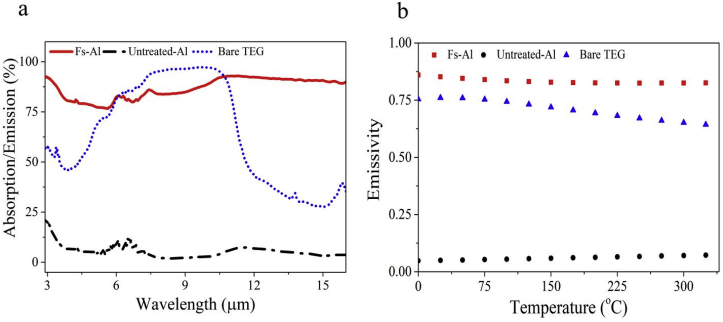
(a) The spectral emission/absorption of untreated-Al (black), TEG (blue) and laser-treated Al (red). The absorption/emission of fs-laser-treated Al is enhanced due to presence of surface structures. (b) The calculated emissivity of the untreated-Al (black dots), bare TEG (blue triangles) and laser-treated Al (red squares) at the temperature range of 0–340 °C. (For interpretation of the references to color in this figure legend, the reader is referred to the Web version of this article.)

To evaluate the effect of fs-laser processing on the thermal emission properties of the treated surface, we measure the spectral emissivity ε(λ) of untreated-Al, a bare TEG and the fs-Al (Fig. 3a). On average, the spectral emissivity of Al experienced ~700% increase upon fs-laser processing. This means that the radiative cooling power $A\sigma \varepsilon \left(T_{c}^{4}-T_{amb}^{4}\right)$ of treated Al increased by 21-fold. Fig. 3b shows the calculated emissivity $\bar{\varepsilon }\left(T\right)$ given by Refs. [27]:

(2) $$\bar{\varepsilon }\left(T\right)=\frac{\int_{0}^{\infty }d\lambda \varepsilon \left(\lambda \right)/\left\{\lambda^{5}\left[\exp\left(hc/\lambda kT\right)-1\right]\right\}}{\int_{0}^{\infty }d\lambda /\left\{\lambda^{5}\left[\exp\left(hc/\lambda kT\right)-1\right]\right\}}$$

where λ is the wavelength ε(λ) is the spectral emissivity of the absorber/emitter, h is the Plank's constant, c is the speed of light, k is the Boltzmann constant, and T is the absorber temperature, here calculated from 0 to 340 °C. The calculated emissivity of the fs-Al for all temperatures is > 0.80 [Fig. 3b] and remains largely invariant across the temperature range under consideration.

The origin of the strong thermal emissivity of fs-Al is due to the formation of random surface nanostructures and the surface oxidation associated with the laser ablation of Al [28]. The random surface structures increase the absorption of the fs-Al due to the excitation of localized plasmon resonances [29]. The plasmonic resonance of random structures is broad. It can span over the visible, NIR, and IR ranges due to two main reasons [30,31]: (i) The size effect where larger structures exhibit plasmonic resonances at the longer wavelength. Surface structures with random sizes, hence, exhibit resonances across a wide range of wavelengths. (ii) The plasmon hybridization effect where the resonance of interacting plasmonic structures shifts to longer wavelengths [30,31]. Consequently, randomly distributed surface structures have a broad plasmon resonance. In addition, the phonon-polariton resonance of Al_2_O_3_ creates strong absorption in the IR wavelength range (~8 μm −15 μm). The high emissivity of bare TEG is due to the strong thermal emission of the ceramic materials used to thermally insulate the TEG [15,32].

**Fig. 4:**
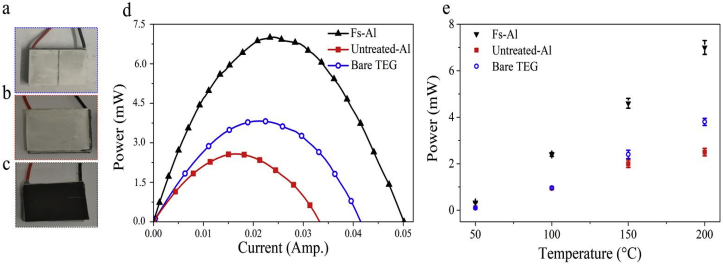
Photographs of (a) a bare TEG with a cold side surface made of ceramics (Bare-TEG), (b) a TEG with an Al foil attached to its back (untreated-Al), and (c) a TEG with fs-laser treated-Al attached to its back (fs-Al). (d) The measured TEG output power vs. current for the three TEGs. (e) The measured peak output TEG power vs. temperature for the systems of the bare-TEG, Al-TEG and fs-treated Al-TEG at different temperatures from 50 °C to 200 °C. Note the improved performance for fs-Al over the entire temperature range under consideration. On the other hand, the TEG output power converge for the bare-TEG and the untreated Al-TEG systems for lower temperatures as radiative cooling becomes insignificant.

To evaluate the performance of fs-Al as a heat exchanger for energy applications, we compare three TEGs: Fig. 4a a bare TEG, Fig. 4b a TEG with an Al substrate attached to its cold side, and Fig. 4c a TEG with fs-Al substrate attached to its cold side. The dimensions of the untreated-Al and fs-Al is the same as the bare TEG (18 mm × 21 mm). Fig. 4d shows the TEG output power measured at 200 °C from the three TEGs. The peak output power for untreated Al, bare TEG, and fs-Al are 2.5 mW, 3.8 mW and 7 mW, respectively (see Supplementary Information, Fig. S1 for more details). Consequently, the TEG output power using a fs-Al heatsink increased by ~280% compared to a conventional Al heatsink.

Fig. 4e shows the temperature dependence of the TEG output peak power for the three systems. At lower temperatures, the output power generated from bare TEG and Al TEG converge and is approximately equal for T ≤ 100 °C. On the other hand, fs-Al TEG has higher power across the entire temperature range under investigation (50°C–200 °C). This is because as $T_{c}$ decreases, the contribution of radiative cooling diminishes, and convective cooling dominates (see supplementary information, Fig. S2). Because fs-Al has a higher surface area, which increases the convective cooling at lower temperatures even when there is no cooling due to thermal radiation. The enhanced convective and radiative cooling of fs-Al makes it ideal for low temperature and high temperature operating conditions.

**Fig. 5:**
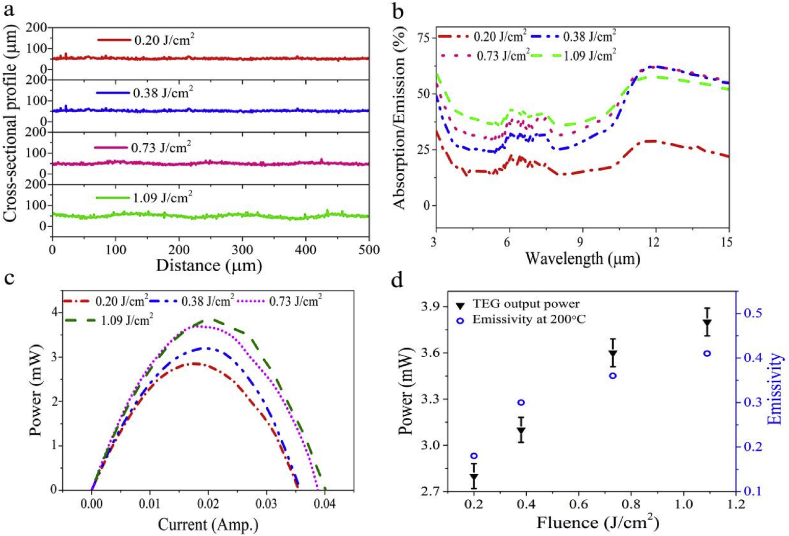
Controlling the absorption/emission of Al by fs-laser-treatment at different laser fluences. (a) Measured grooves depth for fs-Al treated with fluences of *F* = 0.20 J/cm^2^, 0.38 J/cm^2^, 0.73 J/cm^2^ and 1.09 J/cm^2^. (b) The spectral emissivity of the observed nanostructures at the corresponding laser fluences. We see an increase in the emissivity for higher laser fluences. (c) TEG output power for all samples at 200 °C. (d) The maximum TEG output power and emissivity for all the samples at 200 °C, where maximum output power and emissivity is observed at *F* = 1.09 J/cm^2^.

We now study the effect of changing the fs-laser processing parameters on the spectral emissivity and the TEG output power. We processed the Al surfaces to create random surface structures while avoiding the formation of ridges and grooves. Accordingly, we kept the surface area of the treated samples relatively constant while varying the spectral emissivity of the treated samples. To do so, we used a low laser fluence while translating the samples at a relatively fast speed (1.5 mm/s). Fig. 5a shows the obtained depth of created grooves at different laser fluences of *F* = 0.20 J/cm^2^, 0.38 J/cm^2^, 0.73 J/cm^2^ and 1.09 J/cm^2^, respectively.

Fig. 5b shows the measured spectral emissivity at *F* = 0.20 J/cm^2^, 0.38 J/cm^2^, 0.73 J/cm^2^ and 1.09 J/cm^2^, respectively. By increasing the laser fluence, the spectral absorption/emission increases (Fig. 5b). This is due to increased nanoparticle size and density, as we recently demonstrated in Refs. [23]. The surface morphology of the treated samples is shown in the Supplementary Fig. S3, where increased nanoparticle size is observed for higher laser fluence. We perform particle distribution analysis of the samples, which quantifies the particle size dependence on the laser fluence (See Supplementary materials, Fig. S4). As the size and density of nanostructures increase, the randomly distributed, hybridized nanoparticles create broader light absorption as we detailed earlier [23]. By attaching the fabricated fs-Al to the cold side of a TEG, we see an increase in the TEG output power as a function of the surface spectral emissivity, as shown in Fig. 5c. The calculated emissivity and TEG output power for all the laser fluences are shown in Fig. 5d. The calculated emissivity $\bar{\varepsilon }\left(T\right)$ at (200 °C) of fs-Al treated with fluences *F* = 0.20 J/cm^2^, 0.38 J/cm^2^, 0.73 J/cm^2^ and 1.09 J/cm^2^, are 0.18, 0.30, 0.36 and 0.41, respectively (Fig. 5d). The TEG output power generated (Fig. 5c), and emissivity (Fig. 5d), clearly demonstrates the importance of controlling the laser fabrication parameters in optimizing the radiative cooling properties of fs-Al. The maximum TEG power obtained here, however, is lower than that shown in Fig. 4, due to the lower emissivity and surface area of the fabricated samples shown in Fig. 5. Note that creating surface nanostructures via laser ablation without creating micro-grooves will not increase the surface area significantly. From the results obtained in Supplementary Fig. S4, the increase in surface area due to the creation of random surface structures is ~10^−7^ m^2^.

**Fig. 6:**
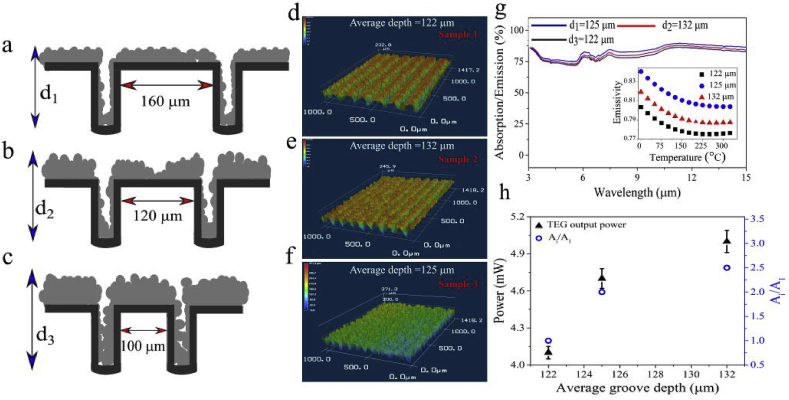
3D profiles and the TEG output power measured from different surfaces created at the same fluence. Fig. 6(a–c) schematically shows the surface area of fs-Al dependence on the grooves interspacing. As we decrease the interspacing distance, the groove depth increases. Consequently, the surface area increases due to increasing the number of the grooves per unit area as well as increasing the grooves depth. Further decrease in the interspacing distance leads to the accumulation of the debris in the grooves which creates shallow grooves with lower surface area. Fig (d–f) are the 3D laser microscopic images obtained at the fluence of *F* = 3 J/cm^2^, where we changed the grooves' interspacing distance from 160 μm, to120 μm and 100 μm, respectively. (g) The spectral emissivity for three different grooves interspacing. The calculated emissivity of three samples at temperature range of 0–340 °C is shown in the inset of (g). (h) The calculated area of three depths and maximum TEG output power, observed for grooves depth of 132 μm.

To study the TEG output power dependence on the fs-Al surface area, we fabricated fs-Al samples with different surface areas while controlling for the emissivity. To do so, we controlled the depth and frequency of the ablated grooves by changing the interspacing between the treated regions while using the same laser fluence. Fig. 6a–c illustrate the mechanism behind optimizing the fs-Al surface area. Laser ablation effectively drills grooves in the treated metal. The debris fall-out covers both the untreated regions forming ridges and the grooves as shown in Fig. 6a, which leads to an overall increase in the surface area. Initially, decreasing the distance between the ablated lines increases the Al surface area by increasing the frequency and the depth of the grooves (Fig. 6b). However, further decreasing the interspacing between ablated lines reduces the surface area due to the accumulation of the debris inside the grooves (Fig. 6c). In other words, in the limit where the interspacing approaches zero, there should be no increase in the surface area except the incremental increase due to the surface roughness caused by laser ablation.

Fig. 6d-f are the 3D laser microscopic images obtained at a laser fluence of *F* = 3 J/cm^2^, where we changed the interspacing between grooves to 160 μm (sample 1), 120 μm (sample 2), 100 μm (sample 3), respectively. The depth of the grooves follows the pattern illustrated in Fig. 6a–c, i.e., it increases as we decrease the interspacing distance (from 160 μm to 120 μm), then decreases by further decreasing the interspacing distance (from 120 μm to 100 μm). The corresponding cross-sectional view for the distance vs depth profiles are shown in the Supplementary Fig. S5. The 3D cross-sectional view allows us to measure the relative change in the fs-Al surface area. The SEM images and TEG output power of the observed nano and microstructures at the corresponding grooves spacing are shown in the Supplementary Fig. S6. The fs-laser-treated area is 11 × 11 mm^2^. The obtained average depths are 122 μm, 125 μm and 132 μm, for sample 1, sample 3, and sample 2, respectively. The spectral emissivity of the three samples are very similar as shown in Fig. 6g. However, the surface area of the treated surfaces differs. Fig. 6h shows the relative increase in the sample surface area defined as $A_{\mathrm{mod}ified}=A_{i}/A_{1}$, where $A_{i}$ is the area of the arbitrary sample and $A_{1}=1$, is the area of the sample 1.$A_{\mathrm{mod}ified}$ is 2 for sample 3 and 2.5 for sample 2. Fig. 6h also shows the maximum output TEG power (T = 200°C) which clearly increases with increasing the sample surface area due to enhanced convective cooling power. It is worth noting that the emissivity of the sample 2 is lower than sample 3 which clearly excludes the small difference in emissivity as the reason behind the observed dependence of the output power on the groove's depth and sample's surface area.

## 4 Conclusion

In summary, we utilized a high-intensity, ultrafast pulsed fs-laser to create nano- and microstructures on Al surfaces for efficient Al-heat exchanger. We optimized for the enhanced spectral emissivity and surface area of the fs-Al, by controlling the laser processing parameters, for instance, fluence, scanning speed, and groove interspacing. For optimum fs-Al heat exchanger, spectral emissivity and surface area is increased by 700% and 300%, respectively. Accordingly, the radiative and convective cooling power of fs-Al is increased by 2100% and 300%, respectively at 200 °C. We used fs-Al as a heat sink on the cold side of the TEG and the combined effect of the enhanced radiative and convective cooling enhanced the temperature difference across the TEG module over a wide temperature range, thus increased its output power to 280% at 200 °C.

Importantly, we showed that enhanced TEG output power from fs-Al persisted even at lower temperatures (<100 °C) due to increase in the convective cooling power. We showed a 330% increase in the TEG output power at 50 °C, when compared with untreated Al heat sink. The technological implications of fs-Al heatsinks are significant. For instance, the average temperature of a processor in a personal computer is ~80 °C. Accordingly, using a commercial black Al heatsink will not increase the cooling power significantly since radiative cooling contributes to <1% of the total cooling power considering a forced convection heat transfer coefficient of ~50. However, using the proposed fs-Al, the cooling power of a heatsink with a given area will increase by 300%. Alternatively, a more compact heatsink can be used.

## Author contributions

M.E. and C.G. discussed and designed the project. S.A.J prepared the samples and performed the laser fabrication. S.A.J, Z. L and Z.Z did AAO deposition. S.A.J. and J.Z performed SEM measurements. S. A. J and S. C. S performed TEG measurements. S. A. J, M.E. performed data analyses. S.A.J, M.E., and C.G. wrote the manuscript. All the authors commented on the paper and discussed the results.

## Declaration of competing interest

The authors declare that they have no conflict of interest.
