# Supplementary material for: Multipronged heat-exchanger based on femtosecond laser-nano/microstructured Aluminum for thermoelectric heat scavengers
Source: Nano Energy. 2020 Sep;75:104987. doi: 10.1016/j.nanoen.2020.104987 (PMC7453914; doi:10.1016/j.nanoen.2020.104987)
Supplement: Multimedia component 1 [file mmc1.docx]

Supplementary information for

**Multipronged heat-exchanger based on femtosecond laser-nano/microstructured Aluminum for thermoelectric heat scavengers**

**TEG output power of the three systems at 50°C, 100°C and 150 °C.**


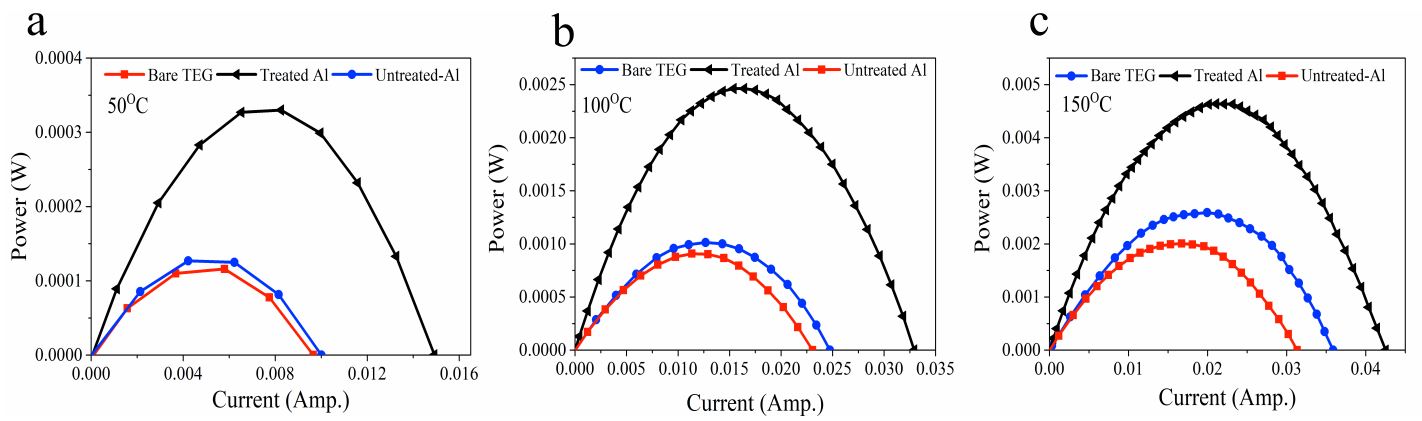


**Figure S1.** The current vs power curves of the three systems a bare-TEG (red lines and squares, Bare-TEG), a TEG with an Al substrate attached to its cold side (blue lines and dots, Al-TEG), and a TEG with fs-Al substrate attached to its cold side (a black line and a triangle), at (a) 50°C, (b)100°C and (c)150°C. The maximum power observed at 50°C for untreated Al, bare TEG and fs-Al is 0.00010 W, 0. 00011 W, and 0.00033 W, respectively. The maximum power observed at 100°C for untreated Al, bare TEG and fs-laser treated Al is 0.00090 W, 0.00111 W, and 0.00241 W, respectively. The maximum power observed at 150°C for untreated Al, bare TEG and fs-laser treated Al is 0.002 W, 0.0024 W, and 0.0046 W, respectively.

**The variation of convective and radiative cooling power density as a function of temperature**





**Figure S2.** The calculated cooling power density of radiative and convective cooling as a function of temperature. Clearly, for low temperatures, convective cooling is dominant. This shows that our multipronged heat exchanger is capable of providing enhanced cooling power at a wide temperature range as it increases both the radiative and convective cooling power.

**The variation of structures density and size as a function of laser fluence.**


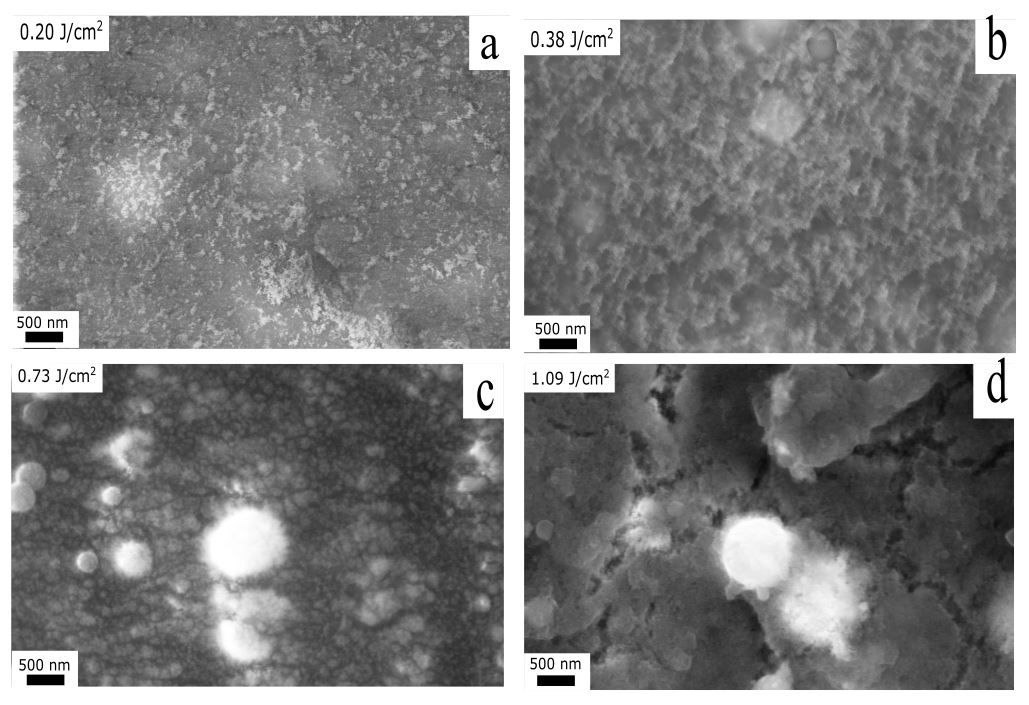


**Figure S3.** (a-d) The surface morphology of the observed nanostructures at the laser fluences *F*= 0.20 J/cm^2^, 0.38 J/cm^2^, 0.73 J/cm^2^ and 1.09 J/cm^2^, respectively, where increasing nanostructures size and density is observed with the increased laser fluence.

**Particle size distribution as a function of the laser fluence**


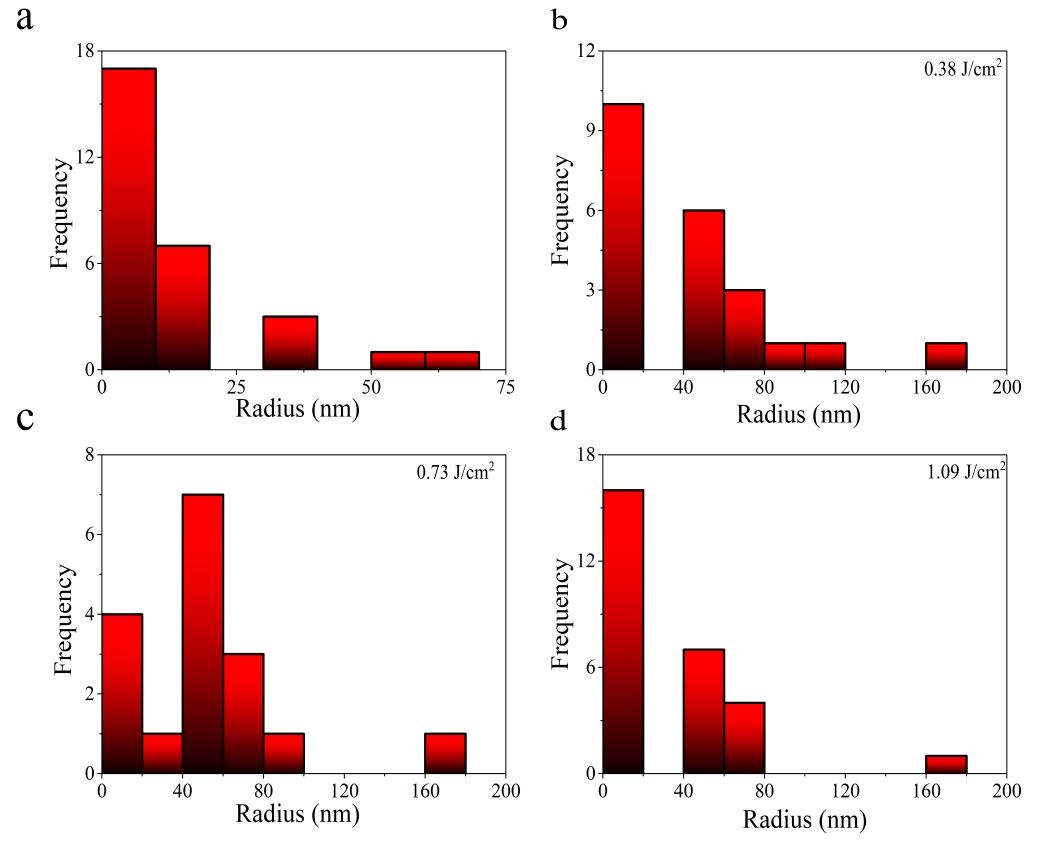


**Figure S4.** Histograms of the size distribution of the formed surface structures at different laser fluences. For **(a)** *F*= 0.20 J/cm^2^, nanoparticles with radius < 70 nm are present. Increasing the laser fluence to **(b)** *F*= 0.38 J/cm^2^ and **(c)** *F*= 0.73 J/cm^2^ creates more surface structures, i.e., higher particle density, and average particle size up to 180 nm. For *F* = 1.09 J/cm^2^ we observe even higher frequency of small and large structures.

**The cross-sectional depth profiles of the grooves as a function of varying interspacing between laser scanned lines.**





**Figure S5.** The cross-sectional view of observed nanostructures at the fluences of *F*=3 J/cm^2^, where we changed the interspacing between grooves to (a) 160 µm, (b) 100 µm and (c) 120 µm, respectively. The corresponding average depths observed are 122 µm, 125 µm and 132 µm, respectively.

**The variation of TEG output power as a function of grooves depth.**


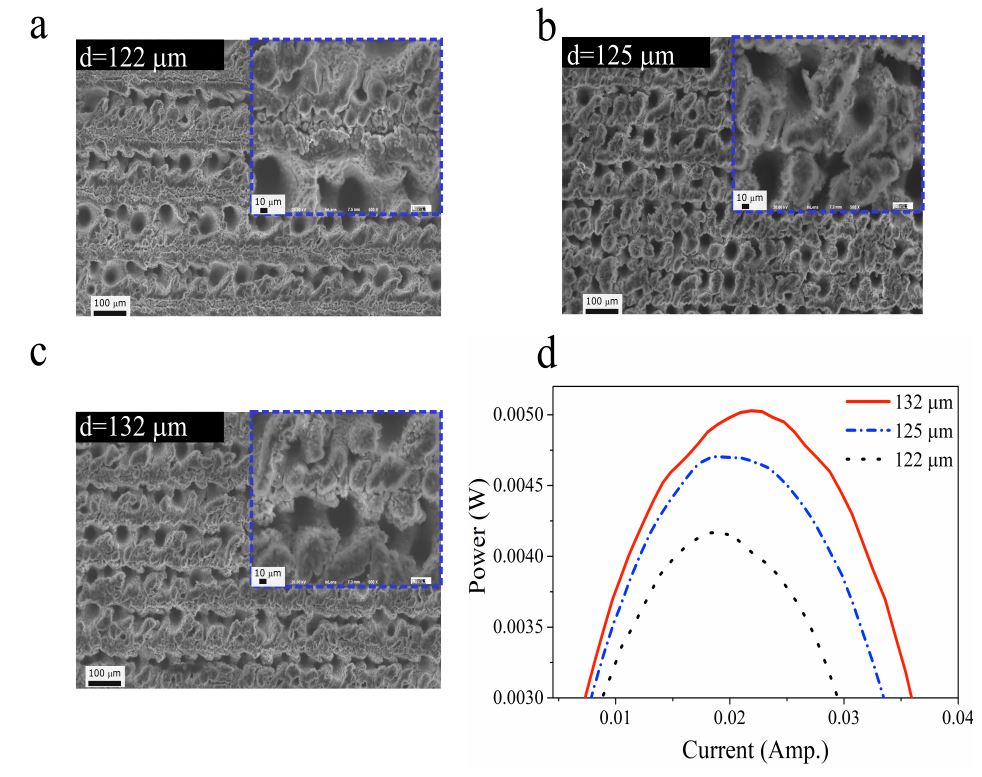


**Figure S6.** (a-c) The SEM images at the fluences of *F*=3 J/cm^2^, where we observed the grooves depth of (a) 122 µm, (b) 125 µm and (c) 132 µm, respectively. (d) The corresponding TEG output power of the three systems where maximum power is obtained for the grooves with maximum depth.
